# A Comparison of the Alken Metallic Telescopic Dilator and Amplatz Serial Dilator for Renal Access in Percutaneous Nephrolithotomy

**DOI:** 10.7759/cureus.72509

**Published:** 2024-10-27

**Authors:** Amitkumar Khwairakpam, Somarendro Khumukcham Singh, Pratik Sharan, Aakash Adhikari, Devendra Singh Mehra, Santosh Yadav

**Affiliations:** 1 Urology, Regional Institute of Medical Sciences, Imphal, IND

**Keywords:** alken metallic dilator, amplatz dilator, intraoperative bleeding, percutaneous nephrolithotomy (pcnl), safety and efficacy

## Abstract

Background: Access to the renal calyx is a challenging and crucial step for a successful percutaneous nephrolithotomy (PCNL). There are different methods of access tract dilatation, and there is no consensus on which method is the best. We conducted a comparative retrospective study on the safety and efficacy of the Alken metallic dilator and Amplatz dilator for renal access in PCNL.

Methods: We retrospectively reviewed the medical records of 80 patients who underwent PCNL between March 2023 and February 2024, and they were divided into two groups. Group A (n=42) comprised patients where Alken dilators were used, while group B (n=38) comprised patients where Amplatz dilators were used. Safety parameters, that is, perioperative bleeding, blood transfusion, access time, postoperative fever, and urosepsis, were compared between the groups. Efficacy in terms of successful renal access and stone clearance was also compared.

Results: The mean access time (mins) was longer in group B than group A (8.1 vs. 7.3, p=0.012). The intraoperative bleeding was more in group B (15.7% vs. 4.7%, p<0.001). Group B had more hemoglobin (g/dl) drop (1.3 vs. 0.7, p<0.001) and need for blood transfusion (18.4% vs. 7.14%, p<0.001) and VAS score (p<0.001) than group A. Postoperative urosepsis was more common in group B. Efficacy in terms of successful renal access and stone clearance was comparable.

Conclusion: The Alken dilator group has a lower rate of blood transfusion and postoperative VAS score. The Amplatz dilator group had more incidences of postoperative urosepsis. The efficacy in both groups was comparable.

## Introduction

Percutaneous nephrolithotomy (PCNL) is the gold standard for treating renal stones of size more than 2 cm, including staghorn calculi [1]. It has replaced open surgeries decades ago due to equal efficacy, less morbidity, and a higher patient acceptance rate [2]. However, bothersome complications of PCNL remain such as bleeding, urosepsis, and urine extravasation [3]. The PCNL procedure is achieved by an initial puncture of a desired calyx under fluoroscopic guidance, followed by dilatation of the nephrostomy tract. This initial step is a crucial and challenging step and the key to success in PCNL. Tract dilatation may be done by using various types of dilators, i.e., serial Amplatz dilators, metallic telescopic Alken dilators, or balloon dilators. It has been previously suggested that blood loss during renal dilation with Amplatz dilators is higher, as with sequential application of fascial dilators, the hemostatic pressure on the parenchyma is lost in between, resulting in intermittent bleeding. On the other hand, telescopic Alken dilators exert a continuous pressure on renal parenchyma; hence, blood loss may be lower [4,5]. Alken dilators are reusable and more durable and hence seem to be more cost-effective. However, there is no consensus about which dilatation method is the best [6]. Alken dilators and Amplatz dilators are the two most commonly used dilators in PCNL. So, in our study, we aimed to compare these two methods of tract dilatations in terms of efficacy and safety.

## Materials and methods

This study was conducted at the Regional Institute of Medical Sciences in Imphal, Manipur, India. As a protocol in our institute, ethical clearance is waived for retrospective study.

We reviewed the medical records of 120 patients who underwent PCNL between March 2023 and February 2024. Patients with previous renal surgery, coagulation disorders, multiple procedures along with PCNL at the same time, PCNL with multiple tracts, and an active urinary tract infection were excluded from the study. A total of 80 patients were eligible for the study. Patients were divided into two groups based on the methods of renal access tract dilatation: group A comprised patients who underwent tract dilatation by an Alken metallic telescopic dilator (n=42), while group B comprised patients who underwent tract dilatation by Amplatz serial dilators (n=38). Both procedures were conducted by two experienced endourologists. We retrospectively assessed the efficacy and safety of PCNL with both methods. As a departmental protocol, we assessed the efficacy of PCNL in terms of stone clearance by using ultrasonography and X-ray of the kidney, ureter, and bladder (KUB) one month after the procedures. Descriptive data like age, gender, stone size, stone location, hardness in Hounsfield unit (HU), punctured calyx, supracostal or infracostal puncture, and number of tracts were noted. Other variables, for example, access time, any access failure, hemoglobin drop and need for blood transfusion, postoperative Visual Analog Scale (VAS), duration of hospital stay, and other complications, were also noted.

Surgical technique

All procedures were done under spinal anesthesia. We gave prophylactic antibiotics according to the local guidelines of the hospital just before giving spinal anesthesia. After placing the patient in the lithotomy position, retrograde insertion of a 5-Fr ureteric catheter into the appropriate kidney was performed under fluoroscopic guidance using a rigid cystoscope. All other parts of the procedure were done in the prone position. We did contrast pyelography through the ureteric catheter, and the desired calyx was punctured by an 18-G initial puncture needle under fluoroscopy guidance. After confirmation of puncture, a terumo 0.035-inch guidewire was inserted, and dilatation of the tract was started. In group A patients, an Alken trocar cannula was inserted into the pelvicalyceal system (PCS), followed by the removal of the trocar and insertion of a guide rod with a distal bulb. After that, the Alken cannula was taken out, and over the guide rod, telescopic Alken dilators were inserted one over another, starting from 12 Fr up to 24 Fr. At the end of dilatation, a 24-Fr Amplatz sheath was placed and a nephroscope inserted. Access time was noted from the initial puncture to the placement of the Amplatz sheath. In group B patients, after the initial calyceal puncture, the tract was sequentially dilated over the guidewire using fascial dilators up to 10 F, and then an 8-Fr cobra dilator was inserted. Over the cobra dilator, subsequent dilators of sizes from 12 Fr up to 24 Fr were inserted, and at the end, a 24-Fr Amplatz sheath was placed and a nephroscope inserted. Stones were fragmented using pneumatic lithoclast. Access time was noted. After completing the procedure, either a 5-Fr DJ stent or a 20-Fr nephrostomy tube was placed, and standard postoperative care of management was followed. After the procedure, for analgesia, we gave a paracetamol infusion of 1 g every eight hours and injection tramadol 100 mg IV on demand.

Statistical analysis

All descriptive values were mentioned in terms of mean and standard deviation. For analytical values, Student's t-test was used to compare between continuous variables, and the chi-squared test was used to compare between categorical variables. A significant p-value will be considered if <0.05. The VAS was used to measure subjective pain scores postoperatively. All analysis was done using IBM SPSS Statistics for Windows, Version 22.0 (Released 2013; IBM Corp., Armonk, New York, United States).

## Results

The two groups were similar in terms of age and sex, and there were no significant differences in preoperative variables such as body mass index (BMI), number of patients with previous urinary tract infections, stone laterality, and mean stone sizes (Table 1).

**Table 1:** Preoperative characteristics of the analyzed group of patients BMI: body mass index; SD: standard deviation; ^1^: Student's t-test (two-tailed); ^2^: chi-squared test p<0.05 is considered statistically significant

Variable			Alken telescopic dilators (n=42) (group A)	Amplatz serial dilators (n=38) (group B)	t-value/χ2-value	p-value
Mean age in years (range)			42.76 (23-69)	46.46 (25-71)	t=1.42	0.15^1^
Male, N (%)			20 (47.61%)	18 (47.3%)	χ2=1.42	0.98^2^
Female, N (%)			22 (52.3%)	20 (52.6%)		
BMI (mean±SD)			24.74±1.64	24.53±1.55	t=0.56	0.57^1^
Previous infection (n, %)			6 (14.28)	7 (18.42)	χ2=0.61	0.76^2^
Stone location, N (%)	Upper calyx		2 (4.76%)	1 (2.63%)	χ2=0.46	0.97^2^
	Middle calyx		8 (19.04%)	7 (18.42%)		
	Lower calyx		8 (19.04%)	8 (21.05%)		
	Pelvis		18 (42.85%)	17 (44.7%)		
	Staghorn		6 (14.28%)	5 (13.15%)		
Stone size (mean±SD) (mm)			21±10	20±8	t=1.21	0.41^1^
Stone side		(Right/left)	19/23	17/21	χ2=0.002	0.96^2^

Intraoperative variables

When compared between both groups, there were no significant differences in the types of entrance calyx (upper/middle/lower), site of access (supracostal/infracostal), or access failure rate. But the mean access time (mins) was longer in group B than group A (8.32 vs. 7.43), which was statistically significant (p=0.012). The intraoperative bleeding was more in group B (21.05% vs. 4.76%, p=0.041). The intraoperative variables are summarized in Table 2.

**Table 2:** Intraoperative characteristics of the analyzed group of patients SD: standard deviation; ^*^: statistically significant; ^1^: Student's t-test (two-tailed); ^2^: chi-squared test p<0.05 is considered statistically significant

Variable		Alken telescopic dilators (n=42) (group A)	Amplatz serial dilators (n=38) (group B)	t-value/χ2-value	p-value
Access time (mean±SD) (min)		7.43±1.11	8.32±1.21	t=3.42	0.012*^1^
Access failure, n		0	0		
Entrance calyx, N (%)	Upper	9 (21.4%)	7 (18.42%)	χ2=0.147	0.92^2^
	Middle	7 (16.66%)	7 (18.4%)		
	Lower	26 (61.90%)	24 (63.15%)		
Supracostal puncture, N (%)		7 (16.6%)	3 (7.89%)	χ2=1.404	0.23^2^
Infracostal puncture, N (%)		35 (83.3%)	35 (92.1%)		
Intraoperative bleeding, N (%)		2 (4.76%)	8 (21.05%)	χ2=4.841	0.041*^2^

Postoperative variables

The stone clearance and mean hospital stay were similar in both groups (p>0.05). Group B had more hemoglobin (g/dl) drop (0.99.3 vs. 0.71, p=0.045), need for blood transfusion (23.6% vs. 7.14%, p=0.039), and postoperative VAS score than group A (p<0.001). Ten patients in group B had postoperative sepsis while none in group A (10 vs. 0, p=0.041). The postoperative variables are summarized in Table 3.

**Table 3:** Postoperative characteristics of the analyzed group of patients SD: standard deviation; ^*^: statistically significant; ^1^: Student's t-test (two-tailed); ^2^: chi-squared test p<0.05 is considered statistically significant

Variable		Alken telescopic dilators (n=42) (group A)	Amplatz serial dilators (n=38) (group B)	t-value/χ2-value	p-value
Hemoglobin) drop (mean±SD) (g/dl)		0.71±0.53	0.992±0.65	t=0.040	0.045*^1^
Need for blood transfusion, N (%)		3 (7.14%)	9 (23.6%)	χ2=4.281	0.039*^2^
Postoperative VAS score (mean±SD)	Day 0	4.90±1.055	6.79±1.27	t=7.22	<0.001*^1^
	Day 1	4.11±0.91	5.6±0.85	t=7.47	<0.001*^1^
Hospital stay (mean±SD) (days)		5.17±0.88	5.47±1.91	t=0.92	0.36^1^
Postoperative fever, N (%)		5 (11.9%)	11 (28.9%)	χ2=4.9	0.032*^2^
Postoperative bleeding requiring additional procedure, N		0	0		
Postoperative sepsis, N (%)		0	4 (10.8%)	χ2=4.65	0.041*^2^
Stone clearance, N (%)		36 (90%)	35 (87.5%)	χ2=0.816	0.487^2^

## Discussion

PCNL is the gold standard for the management of renal stones and is still undergoing several modifications. Every step during PCNL is of paramount importance; however, calyceal puncture and dilatation of the tract are the most crucial steps. Two common dilators used are Alken dilators and Amplatz dilators [7]. Urosepsis and bleeding are the two most feared complications of PCNL. Bleeding depends on various factors like methods of tract dilatation, parenchymal thickness, number of punctures, and number of tracts. Overshooting during dilatation of the access tract is also another cause of venous bleeding during PCNL. Bleeding in multi-step sequential dilatation is thought to be higher as the tamponading effect of the dilator on the parenchyma is lost during the exchange of dilators. But how much it reflects clinically is not known. In our study, we found that mean access time was significantly longer in group B than group A. Ozok et al. also found similar findings in their study [8]. An increase in the access time increases the radiation exposure, which is again another major concern for the surgeons. Group B had more patients with intraoperative bleeding (defined by the need for intraoperative blood transfusion and hemoglobin drop >2 g/dl). Intraoperative bleeding hampers visibility, making PCNL difficult, and also increases the operative time. An increase in irrigating fluid pressure for better vision will in turn be likely to increase the incidences of urosepsis. Independent of the dilation technique used, the incidence of blood transfusion is reported to vary between 1% and 45% in the literature [9]. Overall blood transfusion rate in our study was 15%, with significant differences between the groups (group B: 23.6% and group A: 7.14%). In a study comparing the Alken metallic dilation and Amplatz dilation by Ozok et al., the need for blood transfusion was greater in the Amplatz technique than the Alken dilation, even though it was not statistically significant [8]. In contrast, in the study done by Hijazi et al., they reported no significant difference in access time or hemoglobin drop transfusion rates in both types of dilatation [10]. Bryniarski et al. also found that there is no difference in blood transfusion rate and access time in the Alken dilatation and Amplatz dilatation [11]. Another source of concern for the patients apart from sepsis and bleeding are postoperative pain (VAS score) and compliance. The postoperative pain score was significantly better in group B, thereby likely to increase patient compliance. We also found that the incidence of postoperative fever and urosepsis was more common in the Amplatz dilatation technique. However, there may be multiple confounding factors like a previous positive urine culture, associated comorbidities, excessive manipulation of PCS, supracostal puncture, or non-infective causes like deep vein thrombosis (DVT) or allergic drug reaction. According to the study by Bryniarski et al., postoperative fever and urosepsis were more commonly seen in the Alken dilatation technique, which was contradictory to the findings in our study [11]. We hypothesized that higher irrigant pressure to overcome the poor visibility due to intraoperative bleeding was an important reason for more incidence of urosepsis in group B (Amplatz dilators). The mean postoperative hospital stay and efficacy of both techniques in terms of successful renal access and stone clearance were similar in both groups, which is consistent with other studies reports [8,10,11]. So, based on our study, the Alken dilation method was superior to the Amplatz dilation in terms of safety. However, we need randomized controlled trials with larger sample sizes to validate our findings in the study.

Our study has several limitations, such as a retrospective nature and smaller sample sizes. We have not done proper matching of other confounding factors for urosepsis, such as patients with diabetes mellitus or suffering from other immune deficiency diseases, previous positive urine culture, etc., that increase the risk of urosepsis.

## Conclusions

According to our study, renal access using Amplatz dilators caused more intraoperative bleeding and had a higher blood transfusion rate than metallic telescopic dilatation in PCNL. The Alken metallic telescopic dilatation technique was associated with lesser access time (thus less radiation exposure) and a lower rate of urosepsis. Also, it was associated with less postoperative pain, thereby likely to increase patient compliance. We also observed that both access dilatation techniques had comparable efficacy in terms of successful renal access and stone clearance. However, randomized controlled studies with larger sample sizes are needed to validate our study findings.
